# Conservative Versus Surgical Management of Acute Appendicitis: A Systematic Review

**DOI:** 10.7759/cureus.52697

**Published:** 2024-01-21

**Authors:** Xinlin Chin, Sachini Mallika Arachchige, Jane L Orbell-Smith, Daniela Da Rocha, Anil Gandhi

**Affiliations:** 1 General Surgery, Mackay Base Hospital, Mackay, AUS; 2 Medicine, James Cook University, Mackay, AUS; 3 Medicine and Dentistry, Griffith University, Birtinya, AUS; 4 General Surgery, Canberra Hospital, Australian Capital Territory (ACT) Health, Canberra, AUS; 5 Surgery, Metro North Health, Caboolture Hospital, Caboolture, AUS; 6 General Surgery, Cairns Base Hospital, Cairns, AUS; 7 General Surgery, Monash University, Faculty of Medicine, Nursing and Health Sciences, Selangor, MYS

**Keywords:** uncomplicated acute appendicitis, systematic review, appendicectomy, antibiotics, conservative treatment, acute appendicitis

## Abstract

Recent studies have discussed the role of antibiotic treatment in the conservative management of acute appendicitis and whether antibiotics are a safe option to replace appendicectomy, which has been the gold standard treatment of acute appendicitis for many years. The bibliographic databases Cumulative Index to Nursing and Allied Health Literature (CINAHL), Cochrane, Embase, Medline, and PubMed comparing conservative versus surgical treatment of acute appendicitis were systematically searched according to the Preferred Reporting Items for Systematic Reviews and Meta-Analyses guidelines. Twenty-one studies consisting of systematic reviews and meta-analyses involving 44,699 participants were identified. At least 17,865 participants were treated with antibiotics. Our studies compare antibiotic versus appendicectomy among acute appendicitis patients ranging from 7 to 94 years of age. In most studies, patients received parenteral antibiotics for a total of one to three days, and oral antibiotics such as oral cephalosporin plus metronidazole, oral amoxicillin/clavulanate, oral fluoroquinolones plus Tinidazole upon hospital discharge for a total of 7 to 10 days. The total course of antibiotics for both parenteral and oral regimes ranged from 2 to 16 days, with 10 days being the commonest duration. The recurrence rate following initial antibiotic treatment at one-year follow-up ranged from 13% to 38%, while the mean duration of recurrence ranged from three to eight months. The majority of the patients with recurrence underwent appendicectomy, while some patients were either given a repeat or different course of antibiotics due to the possible presence of antibiotic resistance; however, only 2.4% of the patients were successfully treated upon completion of the second course of antibiotics. Most of the studies concluded that appendicectomy remains the gold standard treatment for uncomplicated acute appendicitis, given its higher efficacy and lower complication rates. Although antibiotic treatment cannot be routinely recommended, it can be considered an appropriate alternative in selected patients with uncomplicated appendicitis who wish to avoid surgery and also acknowledge the risk of recurrence and the potential need for subsequent surgery at the same time.

## Introduction and background

Patients with acute abdominal pain represented 10-20% of the ED presentations [1]. Acute appendicitis was the second most common cause of acute abdominal pain (11-23%) after nonspecific abdominal pain (31-37%) [1]. It is not only the most common emergency abdominal surgery, with a lifetime appendicectomy risk of 23% for females and 12% for males, but also the most frequent cause of intra-abdominal infections, as confirmed by the WISS study [2, 3].

According to a study conducted in New South Wales, Australia, between January 2002 and December 2013 by Schneuer et al., the prevalence of patients who underwent appendicectomy for appendicitis, as well as the proportion of uncomplicated cases, increased with age [4]. Interestingly, the incidence of acute appendicitis is variable - it is stable in most Western countries but appears to be increasing rapidly in newly industrialized countries [3, 5]. This condition, which was once supposed to be rare in many countries of the world, appears to be rising in low- and middle-income countries and in many urban regions [3]. This perhaps may be attributed to changes in diet and lifestyle [3]. On the contrary, many regions of Asia, Latin America, and sub-Saharan Africa are generally thought to have low rates of acute appendicitis [3]. However, it is noted that the true incidence of this condition is unknown in many countries due to unreliable population census, inadequate data, and poor medical record keeping [3, 5].

The Comparison of Outcomes of Antibiotic Drugs and Appendectomy (CODA) trial published in 2020 is, to date, the largest study (N=1552) designed to compare antibiotic treatment with appendicectomy in adults with appendicitis, including those with an appendicolith [6]. This study concluded that antibiotics were non-inferior to appendicectomy, but patients with an appendicolith were at higher risk of undergoing appendicectomy and developing complications compared to those without an appendicolith [6].

Acute appendicitis is a clinical diagnosis based on history, physical examination, laboratory investigations, and imaging [7]. The diagnosis is established in approximately 90% of patients presenting with classic symptoms of appendicitis, which include migratory pain to the right lower quadrant, vague periumbilical pain, low-grade fever, anorexia, nausea, and vomiting [7]. Although historically appendicectomy is the goal standard treatment for acute appendicitis, recently, there has been a marked increase in using broad-spectrum antibiotics as a safe primary approach for patients with uncomplicated acute appendicitis who wish to avoid surgery and the potential postoperative complications [2, 3].

The purpose of this systematic review is to analyze the published evidence to identify the best approach to treat patients with acute appendicitis by comparing the relative effectiveness and outcomes of appendicectomy with non-operative treatment.

## Review

Methods

This systematic review was conducted according to Preferred Reporting Items for Systematic Reviews and Meta-Analysis (PRISMA). The research question driving the review was: is conservative management a better option compared to appendicectomy in acute appendicitis?

Eligibility Criteria

The literature search strategy was created based on the following eligibility criteria: English language articles published from 2012 to 2022. The inclusion criteria for the systematic review were reported discussions of surgery versus conservative treatment (including antibiotics) for acute appendicitis.

Search Strategy

The search strategy was created as ((appendicitis AND appendectomy) AND (conservative treatment) AND review), and the search was undertaken by the professional health librarian team author in January 2022. The search strategy was modified to fit Embase.

Databases Interrogated

The bibliographic databases Cumulative Index to Nursing and Allied Health Literature (CINAHL), Cochrane, Embase, Medline, and PubMed were systematically searched. Duplicates occurring in both databases were removed, and refinement to the topic was undertaken, after which the title and abstract of the records were independently screened.

Data Collection and Data Items

The 21 studies included in the systematic review were analyzed to compare the treatment efficacy of antibiotics versus appendicectomy in acute appendicitis. The identified variables were then extracted from the included studies. The collected study data were number of participants, year of publication, and statistical methods. The extracted outcome variables include antibiotic regime, total duration of antibiotic course, antibiotic treatment efficacy, success rate and recurrence rate of antibiotic treatment, mean duration of recurrence, subsequent treatment needed following recurrence; complications, length of hospital stay, and time to return to work for both antibiotic treatment and appendicectomy.

Results

Search Results

**Figure 1:**
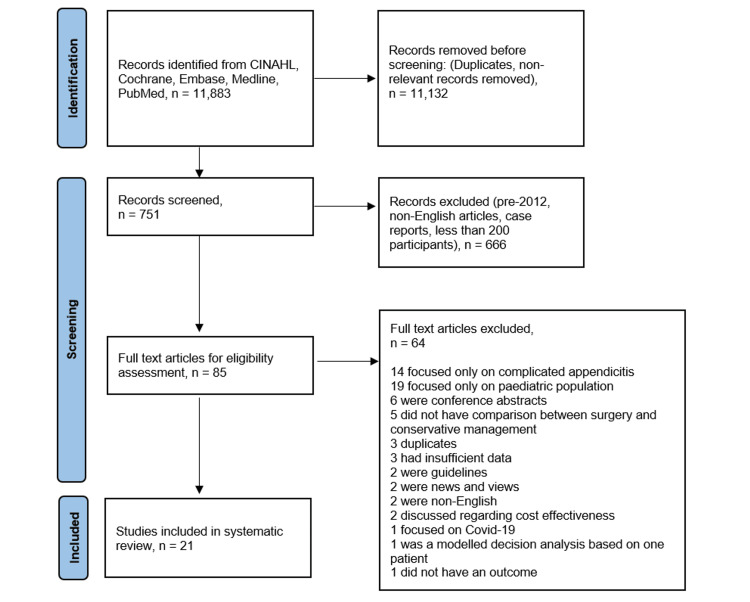
Preferred Reporting Items for Systematic Reviews and Meta-Analyses (PRISMA) flow chart showing methods for article selection

Results of the literature search identified 751 papers for title and abstract screening; of those, 85 papers were identified for full-text assessment. A further 64 papers were excluded, leaving 21 papers for inclusion in the systematic review (Figure 1).

Our systematic review included 21 studies with a total of 44,699 patients [8-28] (Table 1). At least 17,865 participants were treated with antibiotics. Studies involving only the pediatric population were excluded. The 21 studies consist of 16 meta-analyses, three systematic reviews, one cross-sectional survey, and one evidence-based review.

**Table 1:** Summary of antibiotics used upon initial admission and discharge IV - Intravenous; BW - body weight; PO - per os; BD - twice a day; TDS - three times a day; QID - four times a day

Author, Year	Sample size	Age	Initial antibiotics regime	Duration (days)	Antibiotics upon discharge	Total duration (days)
Mason et al., 2012 [10]	Total: 980; Antibiotics: 510; Appendectomy: 470	17-94	1) IV cefotaxime 2g BD + IV tinidazole 80mg QID 2 days, 2) IV cefotaxime 1g BD + IV metronidazole 500mg or 15mg/kg 1 day, 3) IV ciprofloxacin 500mg BD + IV metronidazole 500mg TDS 2 days, 4) IV amoxicillin clavulanic acid 3g per day for BW<90kg; 4g per day for BW >90kg IV or oral 8days depending on the presence of nausea vomiting	1-2	1) Oral ofloxacin 200mg BD + tinidazole 500mg BD for 8 days, 2) ciprofloxacin 500mg BD + metronidazole 400mg TDS, 3) ciprofloxacin 500mg BD + tinidazole 600mg BD for 5 days, 4) amoxicillin plus clavulanic acid 8 days in total	7-10
Varadhan et al., 2012 [12]	Total: 900; Antibiotics: 470; Appendectomy: 430	-	1) IV or oral amoxicillin plus clavulanic acid 3g <90kg or 4g >90kg 48 hours, 2) cefotaxime 1g BD + metronidazole 24 hrs, 3) cefotaxime 2g 12 hourly + tinidazole 800mg daily 2 days	1-2	1) Oral amoxicillin plus clavulinuc acid for total 8 days, 2) Oral ciprofloxacin 500mg BD + metronidazole 400mg tds 10days, 3) Oral ofloxacin 200mg BD + tinidazole 500mg BD for 8-10 days	9-11
Kao et al., 2013 [8]	Total: 1942; Antibiotics: 1591; Appendectomy: 351	> 18	Tinidazole (duration not mentioned)	-	-	-
Liu et al., 2014 [9]	Total: 983; Antibiotics: 391; Appendectomy: 592	27.8 - 38.3	-	-	-	-
Rocha et al., 2015 [11]	Total: 862; Antibiotics: 403; Appendectomy: 458	>18	-	-	-	-
Kirby et al., 2015 [15]	Total: 531; Antibiotics: 263; Appendectomy: 268	-	IV cefotaxime 2g 12 hourly + IV tinidazole 800mg daily for 2 days/ IV or oral amoxicillin and clavulanic acid 3-4g	2	Oral ofloxacin 200mg BD + tinidazole 500mg BD for 8-10 days/ amoxicillin plus clavulanic acid 3-4g 8-16 days in total	8-16
Ehlers et al., 2016 [13]	Total: 1724	26 - 38	Amoxicillin + clavulanic acid	-	-	-
Harnoss et al., 2016 [14]	Total: 2551; Antibiotics: 1312; Appendectomy: 1239	-	1) Ceftaxime + tinidazole, 2) ampicillin + gentamicin + metronidazole, 3) cefotaxime + metronidazole, 4) amoxicillin plus clavulanic acid (1 study), 5) piperacillin tazobactam, 6) 2nd generation cephalosporin + metronidazole, 7) Ertapenem 1st 3 days (IV antibiotics 24-72 hours)	2-3	1) Ofloxacin + tinidazole 10days, 3) ciprofloxacin + metronidazole, 4) amoxicillin and clavulanic acid 8 days, 5) ciprofloxacin + metronidazole, 6) 2nd generation cephalosporin + metronidazole, 7) Levofolxacin from 3rd-10th days	2- 10
Rollins et al., 2016 [16]	Total: 1430; Antibiotics: 727; Appendectomy: 703	18-75	1) Cefotaxime 2 g 12 hourly and tinidazole 800 mg for 2 days, IV 1-3 days 3 use combination of b lactam and nitroimidazole, 2) cefotaxime 1 g twice daily and metronidazole for at least 24 h, 3) Intravenous or oral amoxicillin plus clavulanic acid (3 g per day if\90 kg or 4 g for patients[90 kg) for 48 h, 4) Single daily dose of intravenous ertapenem sodium (1 g/day) for 3 day	1-3	1) oral ofloxacin 200 mg twice daily and tinidazole 500 mg twice daily for 8-10 days, 2) oral ciprofloxacin 500 mg twice a day and metronidazole 400 mg three times a day for 10 days, 3) Intravenous or oral amoxicillin plus clavulanic acid (3 g per day if\90 kg or 4 g for patients[90 kg) for 48 h, 4) 7 days of oral levofloxacin (500 mg once daily) and metronidazole (500 mg three times daily)	-
Sallinen et al., 2016 [20]	Total: 1116; Antibiotics: 510; Appendectomy: 489	-	1) Amoxicillin plus clavulanic acid 3–4g per day for total 8–15 days IV for nausea and oral for others, 2) IV cefotaxime 2g twice daily + tinidazole 800mg once daily for 2 days, 3) IV ertapenem sodium 1g once daily 3 days, 4) IV cefotaxime 2g twice daily and tinidazole 800mg once daily 2 days, 5) IV meropenem 10mg/kg three times daily + metronidazole 20mg/kg once daily for at least 48h	1-3	1) Amoxicillin plus clavulanic acid 3–4g per day for total 8–15 days IV for nausea and oral for others, 2) ofloxacin 200mg and tinidazole 500mg 8 days, 3) oral levofloxacin 500mg once daily + metronidazole 500mg tds 7 days, 4) oral ofloxacin 200mg + tinidazole 500mg BD 10 days, 5) ciprofloxacin 20mg/kg twice daily + metronidazole 20mg/kg once daily 8 days	8-10
Huston et al., 2017 [17]	Total: 530; Antibiotics: 257; Appendectomy: 273	18 - 60	1) IV ertapenem 1g daily 3 days, 2) IV cefotaxime+tinidazole 2 days, 3) IV amoxicillin plus clavulanic acid 2 days	1-3	1) PO levofloxacin 500mg daily + metronidazole 500mg TDS 7 days, 2) PO ofloxacin + tinidazole 10 days, 3) amoxicillin plus clavulanic acid PO 8 days	10-12
Podda et al., 2017 [18]	Total: 1351; Antibiotics: 632; Appendectomy: 719	-	1) IV cefotaxime 2g 12H + IV tinidazole 0.8g daily 2 days, 2) IV ampicillin 1g QID + IV gentamicin 160mg daily + IV metronidazole 500mg TDS, 3) IV amoxicillin plus clavulanic acid 3g (weight <90kg) 4g for weight >90kg if nausea present, otherwise oral for 8days, 4) IV ertapenem sodium 1g daily 3 days	2-3	1) PO ofloxacin 200mg BD + tnidazole 500mg BD 8-10 days, 4) PO levofloxacin 500mg daily + metronidazole 500mg TDS 7 days	10-12
Sakran et al., 2017 [19]	Total: 1430; Antibiotics: 727; Appendectomy: 703	-	1) 3rd gen cephalosporin + nitromidazole, 2) ertapenem, 3) amoxicillin plus clavulanic acid	-	-	-
Poon et al., 2017 [22]	Total: 1304; Antibiotics: 874; Appendectomy: 930	-	1) IV cefotaxime 2 g BD + tinidazole 800 mg daily for 2 days, 2) IV cefotaxime 2 days + tinidazole 800mg daily, 3) IV cefotaxime 1g BD + metronidazole 1.5g q24h for 1 day, 4) IV ciprofloxacin 500mg BD + metronidazole 500mg TDS for 2 days, 5) IV ampicillin 1 g QID + gentamicin 160 mg daily + metronidazole 500 mg TDS for 3 days, 6) Amoxicillin and clavulanic Acid of 3 g or 4 g, 7) IV ertapenem 1 g daily for 3 days	1- 3	1) Oral ofloxacin 200mg BD + tinidazole 500mg BD for 8 days, 2) ofloxacin 200mg, oral ciprofloxacin 500mg BD + metronidazole 400mg TDS 10 days, 4) oral ciprofloxacin 500mg BD + tinidazole 600mg BD 7 days, 5) oral antibiotics for 10 days, 7) oral levofloxacin 500 mg daily and metronidazole 500 mg TDS for 7 days	10-13
Talutis et al., 2017 [25]	Total: 2420; Antibiotics: 1222; Appendectomy: 1198	7 - 75	-	1-2	-	4-10
Prechal et al., 2019 [24]	Total: 1430; Antibiotics: 727; Appendectomy: 703	≥ 18	Levofloxacin + metronidazole + ertapenem	-	-	-
Podda et al., 2019 [21]	Total: 3618; Antibiotics: 1743; Appendectomy: 1875	-	-	-	-	-
Poprom et al., 2019 [23]	Total: 2108	18.5 - 38	1) Penicillin, 2) beta-lactam, 3) beta-lactam + penicillin, 4) cephazolin + metronidazole	-	-	-
Yang et al., 2019 [26]	Total: 2751; Antibiotics: 1463; Appendectomy: 1288	-	-	-	-	-
Wang et al., 2021 [28]	Total: 4551; Antibiotics: 1987; Appendectomy: 2469	8.5 – 38	Beta-lactam/beta-lactamase inhibitor combination (9/21; 43%), cephalosporin (7/21; 33%), carbapenem 5/21 (24%)	-	-	-
Wu et al., 2021 [27]	Total: 10187; Antibiotics: 2056; Appendectomy: 8131	28 - 61	Nitroimidazole combined with another medicine in 1,157 (56.3%) cases and monotherapy in 561 (27.3%) cases. Nitroimidazole (1,337; 65%), third-generation cephalosporins (651, 31.7%), beta-lactam/lactamase inhibitors (601; 29.2%)	3-7	-	-

Antibiotics

The 2021 global clinical pathway for intra-abdominal infections (IAI) completed by the World Society of Emergency Surgery (WSES), the Global Alliance for Infections in Surgery (GAIS), the Surgical Infection Society-Europe (SIS-E), the World Surgical Infection Society (WSIS), and the American Association for the Surgery of Trauma (AAST) recommended intravenous (IV) amoxicillin/clavulanate +/- gentamicin or combinations of IV ceftriaxone or cefuroxime or cefotaxime plus metronidazole as the empiric antibiotic treatment for acute appendicitis patients with normal renal function [3]. IV piperacillin/tazobactam +/- gentamicin is recommended for critically ill patients, while IV ciprofloxacin or IV amikacin plus metronidazole is recommended for patients with beta-lactam allergy [3]. The antibiotic regimes used in most studies were consistent with the clinical recommendations. Initial antibiotics prescribed included combinations of IV cephalosporins (ceftriaxone, cefuroxime, cefotaxime) plus IV metronidazole or tinidazole, a single agent of IV amoxicillin/clavulanate, or IV ertapenem [10, 12-20, 22, 23, 28]. Seven studies included different antibiotic regimens such as IV ampicillin plus IV metronidazole plus IV gentamicin, IV carbapenem plus IV metronidazole or IV levofloxacin, and a single agent of IV tinidazole or IV beta-lactam which were not mentioned in the guidelines [8, 14, 18, 20, 22, 23, 28].

In most studies, patients received parenteral antibiotics for a total of one to three days, and oral antibiotics such as oral cephalosporin plus metronidazole, oral amoxicillin/clavulanate, oral fluoroquinolones plus tinidazole upon hospital discharge for a total of 7 to 10 days [10, 12-20, 22, 23, 27, 28]. The total course of antibiotics for both parenteral and oral regimes ranged from 2 to 16 days, with 10 days being the commonest duration [10, 12, 14, 15, 17, 18, 20, 22, 25].

Response to Treatment

The initial antibiotic treatment success rate for acute appendicitis ranged from 48% to 95%, and 80.6% of patients in a study experienced complete resolution of their symptoms in the initial hospital admission [12, 14, 16, 19, 24, 25, 28]. Studies with one-year follow-up showed antibiotic treatment success rates ranging between 60 to 96.8%, while only one study reported a treatment success rate of 83% at two-year follow-up [11, 13, 17, 25, 27] (Table 2).

**Table 2:** Summary of success and recurrence rates of antibiotic treatment, percentage of patients who failed antibiotic treatment requiring surgery, and those requiring a second course of antibiotics

Author, year	Initial success rate (%)	Recurrence rate (%)	Patients needing surgery (%)	Patients needing a second course of antibiotics (%)
Mason et al., 2012 [10]	-	13%	-	-
Varadhan et al., 2012 [12]	78%	20%	15%	4%
Kao et al., 2013 [8]	-	-	52.5%	-
Liu et al., 2014 [9]	-			
Rocha et al., 2015 [11]	60%	14.2 - 20%	42%	-
Kirby et al., 2015 [15]	-	-	-	-
Ehlers et al., 2016 [13]	-	-	24-35%	-
Harnoss et al., 2016 [14]	68.4%	27.40%	26.5%	-
Rollins et al., 2016 [16]	62.6%	20.40%	97.6%	2.4%
Sallinen et al., 2016 [20]	-	22.6%	8.2%	-
Huston et al., 2017 [17]	72.70%	27.30%	27.30%	-
Podda et al., 2017 [18]	75.90%	22.5%	6.6% had appendectomy in 1st 48hrs, 22.5% in 1st year due to recurrence	-
Sakran et al., 2017 [19]	80%	20.8%	20.80%	-
Poon et al., 2017 [22]	-	13.9-35%	-	-
Talutis et al., 2017 [25]	48-95%	3.6%-36.8%	-	13-22.2%
Prechal et al., 2019 [24]	62.60%	-	5.8% in first admission, 21.4% in subsequent admissions	-
Podda et al., 2019 [21]	67.20%	19.2%	-	-
Poprom et al., 2019 [23]	-	-	-	-
Yang et al., 2019 [26]	-	38%	5.60%	-
Wang et al., 2021 [27]	72.7%	-	-	-
Wu et al., 2021 [28]	-	19.30%	14%	5.3%

While the initial antibiotic treatment success rate appeared promising, initial antibiotic treatment failure rates of 8.5%, 9.2%, and 26.8% among patients with worsening or persistent symptoms requiring appendectomy have been reported [10, 21, 25]. One study reported an overall antibiotic treatment failure rate of 40.2% based on patients who required surgery due to recurrence after the initial hospitalization at the one-year follow-up period [10].

4.9% of the patients who received antibiotic treatment experienced major complications, including 23 cases of appendiceal perforations, one adhesional bowel obstruction, and one death, while seven patients developed minor complications such as superficial wound infection and abdominal discomfort at the one-year follow-up period [20].

Patients who received antibiotic treatment demonstrated reduced analgesia consumption, shorter pain duration, and a lower pain score reported earliest at the 12-hour mark [10, 11, 25]. Two studies showed no significant difference in terms of median pain duration between the antibiotic and the surgical groups and that both groups experienced reduced pain on day six and day 10 [12, 24].

Recurrence

The recurrence rate following initial antibiotic treatment at one-year follow-up ranged from 13% to 38%, while the mean duration of recurrence ranged from three to eight months [10-12, 14, 16, 18-21, 23, 25-27]. Most studies reported patients who received antibiotic treatment in their initial admission underwent surgery upon readmission or recurrence [8, 11-14, 16-18, 20, 24-27]. The majority of patients (42%) who were readmitted and 52.5% of the patients who were initially treated with antibiotics required surgery [8, 11]. Forty-seven out of the 550 patients (pooled estimate 8.2%) with recurrent appendicitis who were initially treated with antibiotics required surgery within a month [20]. Poprom et al. reported the recurrence risk among patients who received a combination of IV cephazolin and IV metronidazole were 36%, 54%, and 86% lower than the IV beta-lactam and penicillin, beta-lactam, and penicillin regime [23].

Seven studies showed that the patients were either given a repeat or different course of antibiotic at readmission or recurrence due to the possible presence of antibiotic resistance; however, only 2.4% of the patients were successfully treated upon completion of the second course of antibiotics [10-13, 16, 25, 27].

Podda et al. reported a possible recurrence rate of 39.1% within five years following the initial course of antibiotic treatment among patients with uncomplicated appendicitis and that 2.3% of the patients with recurrent appendicitis who subsequently underwent surgery were diagnosed with complicated appendicitis [21]. Nonetheless, the antibiotic group showed a significant reduction in the overall complication rate when compared to the appendicectomy group [21].

Discussion

Appendicectomy remains the standard treatment for acute appendicitis with mortality rates of 0.07 to 0.7% and 0.5 to 2.4%, and an overall complication rate between 10 to 19% and 12 to 30% for non-perforated appendicitis and perforated appendicitis respectively [29, 30]. Antibiotic treatment for appendicitis plays an important role in remote areas and is usually the treatment of choice for delayed presentation of appendicitis associated with phlegmon formation [29]. Treating uncomplicated and self-limiting appendicitis with antibiotics may be effective due to the marked reduction in treatment complications and equivalent safety level with appendectomy, but the conservative management of acute appendicitis with antibiotics alone as its initial management approach remains controversial [2, 29]. The usage of antibiotics post-operatively may help to reduce wound infections and intra-abdominal abscesses, but there is little evidence regarding the effectiveness of antibiotics alone in the management of appendicitis and the replacement of appendicectomy with antibiotic treatment [2, 30].

Favors Antibiotics

Patients who underwent appendicectomy generally have a higher complication rate (4.4% to 27%) as compared to patients who received antibiotic treatment (9.1% to 19.4%) and are more prone to developing post-operative complications such as small bowel obstruction, formation of intra-abdominal abscess, wound infection, and wound rupture [9, 13, 16, 25]. 10.8% of the patients who failed antibiotic treatment requiring appendicectomy did not demonstrate a higher rate of complicated appendicitis as compared to the appendicectomy group (17.9%) [16]. Complications secondary to antibiotic treatment include diarrhea, fungal infection, and failure of antibiotic treatment, leading to complicated appendicitis [9, 12]. The success rate of antibiotic treatment ranges from 62.6% in one year up to 83% in two years, while the success rate for surgery is approximately 88.1% at the one-year mark [16].

There is no significant difference in length of hospital stay between patients who were treated with antibiotics and appendicectomy apart from Talutis et al., which showed a longer hospital stay among the antibiotics group (0.4 to 23.2 days) compared to the appendicectomy group (0.83 to 6.4 days) [9, 12, 13, 16, 25]. Patients who underwent surgery required a longer time to return to work due to the longer recovery period when compared to the antibiotics group [9, 13]. Prechal et al. demonstrated a difference of 12 days needed to return to work between the appendicectomy group (19 days) and the antibiotics group (seven days), while Talutis et al. showed only a small difference between the two groups (antibiotic group: 3 to 10 days; appendicectomy group: 5 to 10 days) [24, 25].

Antibiotics appear to be a safe initial treatment approach for most patients with uncomplicated appendicitis as it has fewer complications compared to appendicectomy, and the risk of developing perforation and complications in patients who failed antibiotic treatment requiring a delayed appendicectomy is not increased when compared to the complication rate in the appendicectomy group (10.8% versus 17.9%) [12, 16, 25]. Patients who are managed conservatively with antibiotics need a shorter time to return to work, experience a shorter length of hospital stay, a lower treatment cost, and a lower mortality rate [9, 16]. The therapeutic effect of antibiotics is comparable to appendicectomy; hence, antibiotics appeared as a favorable alternative approach in the treatment of appendicitis [9, 30].

Favors Surgery

Most studies included in our systematic review recommended appendicectomy as the treatment of choice for appendicitis as compared to antibiotics treatment alone [8, 11, 14, 15, 18, 22-24, 27, 28]. Talutis et al. reported 3.6% to 36.8% of the antibiotics treatment group developed recurrence symptoms between 3.4 and 8 months over a follow-up period of 1 to 2 years [25]. Poon et al. demonstrated a recurrence rate of 13.9% to 35% in one year, while three other studies reported an average recurrence rate ranging between 20.8% to 22.6% at the 1-year mark [18-20, 22]. The majority of the antibiotics group who developed symptoms suggestive of recurrent appendicitis underwent appendectomy [11, 25]. 6.6% of the antibiotic treatment group underwent appendicectomy in the first 48 hours, while 22.5% subsequently underwent appendectomy in the first year due to recurrence [18]. Prechal et al. reported similar figures, with 5.8% of the antibiotic group needing appendectomy in the initial admission while 21.4% underwent surgery in their subsequent admissions [24]. Rollins et al. reported out of the 20.4% of the antibiotics group who were readmitted due to recurrent appendicitis, 97.6% had appendectomy while 2.4% were successfully treated with a 2nd course of antibiotics [16]. On average, up to 52.5% of the antibiotic treatment group who developed recurrence required subsequent appendectomies, while 4% to 22.5% were treated with a second course of antibiotics with Mason et al. reported using a different course of antibiotics with the possibility of antibiotic resistance in the initial regime [8, 12, 14, 24, 25, 27]. The length of hospital stay is shorter for patients who underwent surgery, but the time to return to work is longer due to the longer recovery time needed post-operatively as compared to antibiotic treatment [11, 17, 18, 20].

There may be fewer side effects with antibiotic treatment, but the complication rate is higher compared to appendectomy [11, 18, 24, 27, 28]. Harnoss et al. reported a complication rate of 29% in the antibiotics group, where 6.9% of them underwent appendectomy and developed complications [14]. The rate of perforated appendicitis is also higher in the antibiotics group as compared to the appendectomy group (10.6% vs 9.3%) [11]. 10.1% of the antibiotic group developed major complications such as peritonitis, gangrenous, perforated, and complicated appendicitis versus 2.8% of wound infection in the appendectomy group [15]. On the other hand, Poprrom et al. reported although antibiotic treatment demonstrated a lower success rate of 12 to 32%, the risk of complications is about 23-86% lower compared to appendectomy [23]. Surgical complications include infection, obstruction, adhesion, intra-abdominal abscess, re-operation, wound rupture, wound hernia, enterocolitis, enterocutaneous fistula, and bladder injury [18, 23]. Nonetheless, the overall success rate is significantly higher in the appendectomy group [14, 18, 23, 24, 27].

Strengths

Only papers with more than 200 patients were included to improve the quality of the review. Excluding papers before 2012 ensures conclusions are based on contemporaneous data. This systematic review analyses the time to recurrence and the possibility of treating a second episode of appendicitis again with antibiotics.

Limitations

Only English-language articles were included in this systematic review. Non-English articles may be available but not identified or included.

## Conclusions

It has been demonstrated that conservative treatment and surgery in the treatment of appendicitis have their inherent advantages and disadvantages. Hence, decision-making is based on weighing risks and benefits by ensuring the circumstances of each individual are taken into consideration. Although the majority of the studies concluded that appendicectomy remains the gold standard treatment for uncomplicated acute appendicitis given its higher efficacy and lower complication rates, some studies have shown that antibiotic treatment is a safe primary approach for uncomplicated appendicitis with no increased risk of complications even for those who ultimately required surgery after treatment failure with antibiotics. Antibiotic treatment can be considered an appropriate alternative in selected patients with uncomplicated appendicitis who wish to avoid surgery and also acknowledge the risk of recurrence and the potential need for subsequent surgery at the same time.
